# Influence of the development trajectory of interpersonal security on physical activities in school among early adolescents: the mediating effect of control beliefs

**DOI:** 10.3389/fpsyg.2026.1802451

**Published:** 2026-04-10

**Authors:** He Li, Baolin Dong

**Affiliations:** 1 Department of Physical Education, Changchun Finance College, Changchun, Jilin, China; 2 Department of Physical Education, Sanda University, Shanghai, China

**Keywords:** control beliefs, development trajectory, early adolescent, interpersonal security, longitudinal study, physical activities in school

## Abstract

**Objective:**

We explored the development trajectories of interpersonal security among early adolescents and their impact on physical activities in school and examined the mediating effect of control beliefs.

**Methods:**

A whole-year, three-stage follow-up investigation of 2,180 junior high school students (45.32% boys; age, 11.88 ± 1.11 years) from a single province in China was conducted.

**Results:**

For early adolescents, the ideal growth mixture model of the three classifications of interpersonal security was the optimal model (AIC = 14472.50, BIC = 14453.40, aBIC = 14412.11, Entropy = 0.78; LMR: *p* = 0.00, BLRT: *p* = 0.00). When comparing the effects of different development trajectories of interpersonal security on early adolescents’ physical activities in school (T_3_), the relative total influential effects of the high-elevated and medium-sustained groups were 0.22 and 0.26 greater than those of the low-decreasing group. Furthermore, the relative mediating effects of control beliefs were all significant, revealing that, in the high-elevated and medium-sustained groups, and by the mediating effect of controlling beliefs, interpersonal security affected early adolescents’ physical activities in school (T_3_), respectively (*p* < 0.01).

**Conclusion:**

The better the development trajectory of interpersonal security, the easier it is to enhance early adolescents’ control beliefs and their engagement with physical activities in school. Educators and parents should pay more attention to the cultivation of early adolescents’ social adaptability and interpersonal communication skills.

## 1 Introduction

Encouraging adolescents to actively engage in beneficial outdoor activities and improve their physical activity levels in school can help prevent myopia and reduce the risk of developing chronic non-communicable diseases, and this is a globally recognized and effective health intervention for adolescents. In China, multiple surveys indicate that adolescents’ leisure time in school remains dominated by low-intensity, low-energy-expenditure physical activities, with more than 40% of Chinese adolescents still failing to meet the World Health Organization’s recommended standards for healthy activity levels (Ma et al., 2022). It is evident that the physical activity levels of Chinese adolescents during their school years urgently require improvement. Early adolescence—namely, ages 12–15 years—represents a critical period for establishing behavioral habits. Analyzing the underlying mechanisms of physical activity among adolescents during this stage can help uncover various potential issues in their school life and learning experiences. This understanding is essential for developing precise intervention strategies and promoting the healthy physical and mental development of adolescents worldwide.

It is well known that, during early adolescence, individual’s preferred social relationships gradually shift from blood relationships to school interpersonal relationships. This implies that interpersonal development at this stage primarily occurs within the school environment. Research has determined that, when adolescents experienced a sense of security in peer interactions, they were more likely to exhibit positive emotional responses, appropriate coping strategies, and to engage in active behavioral patterns, and they also maintained a state of positive physical engagement (Engels et al., 2002). Interpersonal security is a subcategory of the sense of security, referring to an individual’s experience of maintaining a favorable position regarding their own circumstances within interpersonal relationships (Li et al., 2023).

Attachment theory posits that a child’s early emotional and psychological development often stems from attachment to a specific figure. Secure attachment fosters fewer psychological issues and promotes more positive behaviors. Conversely, a lack of interpersonal security leads to anxious attachment and manifests as negative behavioral patterns in social interactions (Bowlby, 1982; Hazan and Shaver, 1987; Scott et al., 2011). It is undeniable that, despite extensive research confirming that security (including interpersonal security and a sense of assured control) can influence adolescents’ daily behaviors (e.g., smartphone addiction and aggressive behavior) (Lemay and Dudley, 2011), research on the mechanisms through which interpersonal security affects physical activity among early adolescents in school settings remains relatively scarce. Moreover, it is worth noting, and most importantly, that interpersonal security is not a stable, constant experience but rather dynamically changes with the development of an individual’s social cognition and behavior. If we rely solely on cross-sectional surveys to explore the intrinsic connection between interpersonal security and physical activity at school at a single point in time, we may fail to capture the dynamic developmental characteristics of interpersonal security in early adolescence. Furthermore, we may overlook the heterogeneity in how different developmental trajectories of interpersonal security influence physical activities in school during early adolescence. These issues are among the core questions that this study urgently seeks to address.

The core perspective of the social ecological model of health behavior posits that the influence of social environments on physical activity cannot be separated from the mediation of psychological characteristics (Bronfenbrenner and Ceci, 1994; Sallis, 1999). In other words, when examining the impact of interpersonal security on physical activities in school among early adolescents, the mediating role of certain individual psychological factors should be considered. Among these, control beliefs serve as one such factor with this effect. Control beliefs relate to an individual’s understanding of the controllability of external stressors and their perception of the factors influencing that control (Flammer, 1995). First, interpersonal security may determine the locus-of-control beliefs. Cognitive development theory posits that subject experiences triggered by social contexts can reshape cognitive responses (Piaget, 1964)—namely that, as a type of cognitive response, locus-of-control beliefs undergo corresponding adjustments in response to social environmental stimuli (e.g., interpersonal security). Lemay and Dudley (2011) found that a strong sense of interpersonal security fosters high self-esteem and self-confidence, enabling individuals to reduce feelings of helplessness under stressful conditions while enhancing their sense of control and certainty (i.e., control beliefs). Meanwhile, when interpersonal security was lacking (e.g., when experiencing peer rejection), individuals perceived a greater threat to their emotional self-regulation abilities and were more likely to develop low levels of control beliefs (Ajzen and Driver, 1991). In short, a strong sense of interpersonal security enhances an individual’s understanding of self-perception and emotional responses while also improving the controllability and perceptual capacity of beliefs about control (Lepore and Greenberg, 2002). Second, control beliefs serve as a predictor of physical activity among adolescents. The core perspective of the control beliefs theory posits that, as a form of self-emotional management cognition, control beliefs are a crucial cognitive element in adolescent behavioral development, and they can influence individual’s behavioral intentions and performance (Heckhausen and Schulz, 1995). In the field of sports, extensive empirical evidence confirms that control beliefs can predict adolescents’ physical exercise adherence, and they are a prerequisite for adolescents’ physical activity (Dong and Mao, 2018). Based on this, it is inferred that control beliefs mediate the effect of interpersonal security on adolescents’ physical activity in school.

Thus, when considering the dynamic developmental trajectories of interpersonal security, does the influence of different developmental trajectory types of interpersonal security on early adolescents’ physical activities in school exhibit heterogeneity? Could interpersonal security across different developmental trajectory types all indirectly promote early adolescents’ physical activities in school through the control beliefs? Currently, research that answers these questions remains lacking. Therefore, this study primarily investigates early adolescents through a longitudinal cross-year, three-stage tracking survey. Specifically, it explores the heterogeneity in interpersonal security developmental trajectories and their impact on early adolescents’ physical activities in school while also examining the mediating role of control beliefs. The findings obtained from this research could be helpful in preventing a variety of problems in the academic and personal lives of adolescents.

## 2 Materials and methods

### 2.1 Data and participants

Data were collected from 2,940 Chinese adolescents from 12 public and private junior high schools in Jiangsu Province and Shanghai, China, in September 2022 (T_1_), March 2023 (T_2_), and September 2023 (T_3_). Data collection was conducted in classroom settings using paper questionnaires. A total of 498 questionnaires were excluded according to the following criteria for identifying invalid scales: missing data on the frequency or time of physical activity of any intensity, failure of the reverse-scoring item, a response rate of <85%, and responses inconsistent with facts. Eventually, 2,180 valid questionnaires were retained. The age range of the survey participants was 11–13 years (although seven adolescents did not specify their age), with a mean age of 11.88 years (*SD* = 1.12 years), and 45.32% of the sample were men. The numbers of students in grades 6–8 totaled 680, 761, and 739, respectively. The sample size of this analysis meets the social survey sample size standard by using G*Power test. The specific settings were as follows that test family = *χ*^2^ tests, statistical test = Goodness-of-fit tests: Contingency tables, Type of power analysis = A prior: Compute required sample size—given α, power, and effect size, Input paramenters: Effect size w = 0.5, α err orob = 0.01, power (1-β err prob. = 0.95, *df* = 5). Then the total sample size of output parameters was “Not less than 134.” The study protocol was approved by the appropriate ethics committee. Before the survey was administered, written informed consent was obtained from all participants.

During survey analysis, referring to the experience of Loukas et al. (2016), missing values were imputed using the full information maximum likelihood method. Chi-squared and *t* tests revealed no significant differences between the lost and analysis samples in terms of gender (*χ*^2^_(1)_ = 2.62), grade level (*t*_(2)_ = 0.63), T_1_ interpersonal security (*t*_(2,178)_ = −1.15), T_1_ control beliefs (*t*_(2,178)_ = −1.04), T_1_ physical activities in school (*t*_(2,178)_ = −0.45), T_2_ interpersonal security (*t*_(2,178)_ = −1.08), T_2_ control beliefs (*t*_(2,178)_ = −1.18), and T_2_ physical activities in school (*t*_(2,178)_ = −0.60) (*t <* 1.96, *p* > 0.05).

### 2.2 Measures

#### 2.2.1 Interpersonal security

Eight items from the Security Questionnaire by Cong and An (2004) were used to measure interpersonal security. Responses were coded on a 5-point Likert scale ranging from 1 (“Very inconsistent”) to 5 (“Very consistent”) points, with higher scores indicating a greater level of interpersonal security. The respective indicators for exploratory factor analysis and confirmatory factor analysis are shown in Table 1. The stability coefficient of repeated measures was 0.68 (*p* < 0.01).

**Table 1:** Indices of exploratory factor analysis and confirmatory factor analysis about each subscales.

Variable	Exploratory factor analysis					Confirmatory factor analysis								
	KMO	Bartlett-test	*df*	*p*	CCR	*x* ^2^ */df*	*df*	GFI	NFI	IFI	NNFI	CFI	RMSEA	SRMR
IS (T_1_)	0.89	4000.77	28	0.001	55.75	2.49	20	0.95	0.93	0.95	0.91	0.95	0.07	0.05
IS (T_2_)	0.91	5859.18	28	0.001	69.59	2.50	20	0.95	0.96	0.96	0.93	0.96	0.07	0.05
IS (T_3_)	0.91	6167.77	28	0.001	66.89	2.53	20	0.94	0.96	0.97	0.93	0.97	0.07	0.04
CB(T_1_)	0.89	2889.68	15	0.001	61.54	2.16	9	0.98	0.98	0.98	0.97	0.98	0.08	0.03
CB(T_2_)	0.90	3280.25	15	0.001	66.33	2.15	9	0.97	0.97	0.97	0.96	0.97	0.08	0.03
CB(T_3_)	0.91	3912.29	15	0.001	68.54	2.14	9	0.98	0.98	0.96	0.96	0.98	0.08	0.03

CB, control beliefs; IS, interpersonal security; CCR, cumulative contribution rate.

#### 2.2.2 Control beliefs

Six items from the control beliefs subscale of the Adolescent Control, Strategy, and Competence Beliefs Scale by Skinner et al. (1988, 2012) were used to measure control beliefs. Responses were coded on a 5-point Likert scale ranging from 1 (“Never before”) to 5 (“Always”) points, with higher scores indicating a greater level of control beliefs. The respective indicators for exploratory factor analysis and confirmatory factor analysis are shown in Table 1. The stability coefficient of repeated measures was 0.61 (*p* < 0.01).

#### 2.2.3 Physical activity

Seven items from the International Physical Activity Questionnaire—Short Form by Craig et al. (2003) were used to measure physical activity. Among then, the first six items asked participants to recall their physical activity status during the most recent weekdays (Monday through Friday) in school, while the final item assessed participants’ sedentary time during the most recent weekdays (Monday through Friday) in school. The scale assigned metabolic-equivalent values of 3.3 for walking, 4.0 for moderate-intensity physical activities, and 8.0 for vigorous-intensity physical activities, respectively. Following the methodology of Meeus et al. (2011), the data underwent cleaning, truncation, outlier handling, and grouping by physical activity level. The “physical activity level” group was adopted as the assessment metric for physical activity in this study. The stability coefficient of repeated measures was 0.68 (*p* < 0.01).

### 2.3 Measurement process

We conducted specialized online training via Tencent Meeting (Tencent Holdings Ltd., Shenzhen, China) for personnel responsible for data collection (including research team members or homeroom teachers). Consistency in testing procedures and processes was maintained across the three data-collection periods. The questionnaire was administered in the classrooms of each sampled class using a group testing method to collect data. Prior to administering each questionnaire, we obtained consent from the sampled schools, homeroom teachers, and surveyed students. We also verbally explained the instructions, including the purpose of data collection, storage methods, confidentiality regulations, and the voluntary nature of participation. In addition to collecting demographic information (school, gender, grade, age, etc.) from participants during three data-collection sessions, respondents were required to accurately enter the last eight digits of their student ID number for data-coding purposes.

### 2.4 Statistical analyses

First, exploratory factor analysis, confirmatory factor analysis, Spearman rank correlation analysis, and kappa consistency testing were used to assess the reliability and validity of the measurement tool. According to Dong and Mao (2018), Kaiser–Meyer–Olkin (KMO) index greater than 0.8 and Bartlett’s sphericity test reaching statistical significance (*p* < 0.01) indicate acceptable content validity of the data. Furthermore, the χ^2^/*df* ratio was below 5, and fit indices including *GFI*, *NFI*, *IFI*, *NNFI*, and *CFI* exceeded 0.9, and the *RMSEA* remained below 0.8, confirming acceptable construct validity of the data. Additionally, Cronbach’s coefficients exceeding 0.8 and the stability coefficient for repeated measures exceeding 0.5 indicate that the data exhibit good internal consistency reliability and stability reliability. Then, descriptive statistics and correlation analysis were used to assess the relationships among variables. Third, we used Mplus (version 8.3; Muthén and Muthén, Los Angeles, CA, United States) to construct a latent variable mixed growth model to examine the developmental trajectory types of interpersonal security. Following the work of Nylund et al. (2007), this study evaluated the optimal number of classes using the lowest Akaike information criterion (AIC), Bayesian information criterion (BIC), and adjusted BIC (aBIC) values, along with a high entropy value (close to 1.0), with no less than 5% of participants in each class. For study purposes, a significant Lo–Mendell–Rubin (LMR) test result (*p* < 0.05) demonstrates adequate model parsimony, and a significant bootstrapped likelihood ratio test (BLRT) statistic (*p* < 0.05) accepts k-group classification while rejecting *k* − 1 group classification. If the LMR and BLRT results conflicted, we prioritized the BLRT outcome (Nylund et al., 2007). Furthermore, the subgroup proportions of category probabilities was required to exceed 5%. Finally, we used the bootstrap method to examine the relative mediating effects of multiple independent variables. Furthermore, in order to control for the influence of demographic variables, variables such as gender and place of origin, etc. were included as control variables based on the methodology of Dong and Mao (2018).

## 3 Results

The correlation coefficients of the analyzed variables are shown in Table 2. Any two of the interpersonal security of September 2022 (T_1_), March 2023 (T_2_), and September 2023 (T_3_) were significantly positively correlated (*p* < 0.01). The same was true for control beliefs (*p* < 0.01) and physical activities in school (*p* < 0.01). Separately, significant and positive correlations were observed among interpersonal security (T_1_), control beliefs (T_1_), and physical activities in school (T_1_) (*p* < 0.01); among interpersonal security (T_2_), control beliefs (T_2_), and physical activities in school (T_2_) (*p* < 0.01); and among interpersonal security (T_3_), control beliefs (T_3_), and physical activities in school (T_3_) (*p* < 0.01), respectively.

**Table 2:** Correlation analysis of interpersonal security, control beliefs, and physical activities in school.

Variable	*M*	*SD*	(1)	(2)	(3)	(4)	(5)	(6)	(7)	(8)	(9)
(1). IS (T_1_)	29.36	7.19	1								
(2). IS (T_2_)	30.57	7.01	0.49^**^	1							
(3). IS (T_3_)	31.11	7.18	0.48^**^	0.49^**^	1						
(4). CB(T_1_)	22.56	5.37	0.58^**^	0.37^**^	0.30^**^	1					
(5). CB(T_2_)	22.32	5.46	0.43^**^	0.51^**^	0.38^**^	0.50^**^	1				
(6). CB(T_3_)	23.03	5.35	0.40^**^	0.61^**^	0.51^**^	0.51^**^	0.52^**^	1			
(7). PA in school (T_1_)	1.84	0.83	0.22^**^	0.16^**^	0.39^**^	0.24^**^	0.27^**^	0.32^**^	1		
(8). PA in school (T_2_)	1.83	0.76	0.21^**^	0.23^**^	0.37^**^	0.31^**^	0.34^**^	0.41^**^	0.52^**^	1	
(9). PA in school (T_3_)	1.87	0.85	0.18^**^	0.29^**^	0.30^**^	0.42^**^	0.50^**^	0.41^**^	0.51^**^	0.51^**^	1

CB, control beliefs; IS, interpersonal security; PA, physical activities. ^**^P < 0.01.

The results of latent growth mixture modeling concerning early adolescents’ interpersonal security development trajectories are shown in Table 3. Following Nagin (2005), we found that estimating three trajectory classes was optimal: AIC = 14388.47, BIC = 14453.40, aBIC = 14412.11, and Entropy = 0.78. Broadly, 51.24, 5.32, and 43.45% of participants were assigned to each class. Significant LMR (*p* < 0.01) and BLRT (*p* < 0.01) results were noted.

**Table 3:** Fit indicators for the growth mixture model latent class analysis of interpersonal security.

Category	AIC	BIC	aBIC	Entropy	LMR	BLRT	Category probability (%)
1	–	–	–	–	–	–	–
2	14472.50	14522.44	14490.68	0.58	0.05	0.00	29.82/70.18
3^△^	14388.47	14453.40	14412.11	0.78	0.00	0.00	51.24/5.32/43.45
4	14362.68	14442.60	14391.78	0.80	0.24	0.00	42.99/50.41/5.32/1.28
5	14336.82	14431.72	14371.37	0.82	0.90	0.99	4.03/42.81/1.47/1.83/49.86

^△^Indicates the optimal fit model. aBIC, adjusted Bayesian information criteria; AIC, Akaike information criteria; BIC, Bayesian information criteria; BLRT, bootstrapped likelihood ratio test; LMR, Lo–Mendell–Rubin.

**Figure 1:**
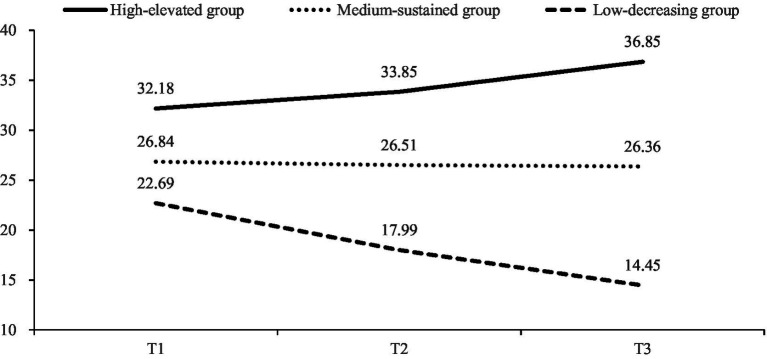
Interpersonal security development trajectory types.

This study identified three development trajectories of early adolescents’ interpersonal security (Figure 1): Class 1, designated as the high-elevated group with a significant intercept (*I* = 32.05, *p* < 0.01) and positive slope (*S* = 4.60, *p* < 0.01), included 112 adolescents (51.24%). The adolescents in this group demonstrated relatively high levels of interpersonal security to begin with, with a subsequent rise in such. Class 2, designated as the medium-sustained group with a significant intercept (*I* = 27.04, *p* < 0.01) and an insignificant negative slope (*S* = −0.34, *p* > 0.05), included 947 adolescents (43.45%). Adolescents in this group showed medium levels of interpersonal security and a stable trend in such after 1 year. Class 3, designated as the low-decreasing group with a significant intercept (*I* = 23.00, *p* < 0.01) and a negative slope (*S* = −7.79, *p* = 0.05), included 116 adolescents (5.32%). Adolescents in this group demonstrated low initial levels of interpersonal security and a downward trend in such within a year.

The results of the multiple categorical independent variable regression analysis are shown in Table 4. This analysis was controlled for demographic variables, control beliefs (T_1_), control beliefs (T_2_), physical activities in school (T_1_), and physical activities in school (T_2_). The type of interpersonal security developmental trajectory was recoded as a dummy variable, with the low-decreasing group used as the reference category. Model 1 shows that the medium-sustained group and the high-elevated group exhibited relatively direct effects on physical activities in school that were 0.22-fold and 0.26-fold greater than that of the low-decreasing group, respectively. These results indicate that, the better the interpersonal security development trajectory of early adolescents, the greater their engagement in physical activities during school activities.

**Table 4:** Relative mediation effect analysis of control beliefs.

Variable	Model 1 PA in school (T_3_)			Model 2 Control beliefs (T_3_)			Model 3 PA in school (T_3_)		
	β	*se*	*P*	β	*se*	*P*	β	*se*	*P*
Control variables									
Place of origin	−0.08^**^	0.05	0.002	−0.09^***^	0.26	0.000	−0.06^*^	0.05	0.020
Age	−0.06	0.03	0.422	0.01	0.19	0.079	−0.06	0.03	0.395
Gender	−0.12^***^	0.05	0.000	−0.05^*^	0.26	0.038	−0.11^***^	0.04	0.000
Grade	−0.01	0.03	0.965	−0.04	0.19	0.553	0.01	0.03	0.911
Control beliefs (T_1_)	0.15^*^	0.01	0.049	0.33^***^	0.03	0.000	−0.04	0.01	0.183
PA in school (T_1_)	0.41^***^	0.03	0.000	0.32^***^	0.16	0.000	0.40^***^	0.03	0.000
Control beliefs (T_2_)	0.16^*^	0.01	0.043	0.34^***^	0.02	0.000	0.18^*^	0.03	0.033
PA in school (T_2_)	0.43^***^	0.04	0.000	0.02	0.01	0.172	0.31^***^	0.03	0.000
Predictor variable									
Medium-sustained group	0.220^**^	0.099	0.009	0.40^***^	0.56	0.000	0.18^*^	0.10	0.050
High-elevated group	0.257^**^	0.101	0.006	0.53^***^	0.58	0.000	0.20^*^	0.10	0.047
Control beliefs (T_3_)							0.28^***^	0.01	0.000

The developmental trajectory type of the “low-decreasing group” of interpersonal security was used as a reference. PA, Physical activities. ^***^P < 0.001, ^**^P < 0.001, ^*^P < 0.05.

In addition, the relative effects of interpersonal security development trajectories on control beliefs 1 year later are presented in Model 2 (Table 4). The developmental trajectory of the medium-sustained and high-elevated groups demonstrated 0.340-fold and 0.53-fold greater relatively direct effects on control beliefs compared to the low-decreasing group. This finding indicates that, the better the interpersonal security developmental trajectory type of early adolescents, the greater their control beliefs.

The predictive results of control beliefs at T_3_ on physical activities in school at T_3_, along with the relative direct and indirect effects of interpersonal security developmental trajectories on T_3_ physical activities in school, are presented in Model 3 (Table 4). Control beliefs at T_3_ significantly influenced physical activities in school at T_2_ (β = 0.28; 95% CI: 0.04, 0.06; *p* < 0.001). After controlling for control beliefs at T_3_ (mediating variable), the relative effects of the medium-sustained group and the high-elevated group on physical activities in school were 0.18-fold greater (β = 0.18; 95% CI: 0.02, 0.38; *p* < 0.05) and 0.20-fold greater (β = 0.20; 95% CI: 0.14, 0.35; *p* < 0.05) than that of the low-decreasing group. These results indicate that control beliefs exhibit a significant relative mediating effect on the interpersonal security developmental trajectory influence on early adolescents’ physical activities in school—namely, the better the interpersonal security developmental trajectory type of early adolescents, the higher their control beliefs, and the more active they are in physical activities during school as a result.

## 4 Discussion

### 4.1 Types and characteristics of the developmental trajectory of early adolescents’ interpersonal security

Our analysis of longitudinal survey data from 2,180 Chinese adolescents aged 11–13 years showed that most early adolescents’ interpersonal security is at a medium-to-high level. Furthermore, the developmental trajectories of interpersonal security exhibit heterogeneity and can be categorized into three distinct types—namely, a high-elevated group, a medium-sustained group, and a low-decreasing group. In our study, 51.24% of the early adolescents who exhibited initial interpersonal security scores at a relatively high level with a continuous rise within 1 year were included in the high-elevated group; meanwhile, 43.45% of the early adolescents who initially had initial interpersonal scores at a moderate level and who showed a stable trend over the course of 1 year were included in the medium-sustained group. This result is consistent with the views of Deng et al. (2022). From childhood through adolescence, children gradually establish broader connections with people outside their families, with peers becoming the primary focus of their social relationships (Sullivan et al., 1953). Moreover, influenced by traditional Confucian thought, adolescents living in China generally embody the principles of “benevolence loves others, righteousness respects others” in their interpersonal relationships—that is, showing mutual respect, mutual understanding, mutual courtesy, and maintaining harmony. Especially during the early stages of adolescence, both group-oriented, unidirectional interpersonal relationships (Acceptance) and individual-oriented, bidirectional interpersonal relationships (Friendship) serve as vital sources for adolescents to maintain self-esteem, shape their personalities, and develop social skills (Sullivan et al., 2017). Therefore, early adolescents, who experience moderate to high levels of interpersonal security during their early years, are more likely to maintain or enhance this sense of security in future social interactions, thereby achieving group identification or gaining peer acceptance. This aligns with Berger’s interpersonal communication theory, which posits that established social recognition, peer acceptance, and other secure social experiences enable early adolescents to develop positive cognitive schemas and self-evaluations and to enrich their social skills and interpersonal experiences, thereby maintaining or enhancing interpersonal security (Berger, 2010).

Of note, 5.32% of included adolescents initially had low levels of interpersonal security and showed a downward trend in such within 1 year (i.e., the low-decreasing group). This result could be understood through the theory of depressive interpersonal relationships, which posits that negative social experiences (e.g., rejection and exclusion) can lead to psychological issues like depression and self-harm. Moreover, as the frequency and intensity of negative social experiences increase, an individual’s tendency toward social withdrawal will also persistently intensify (Stattin and Latina, 2018). It is evident that, for early adolescents, experiencing insecurity in interpersonal interactions (e.g., rejection, exclusion, or refusal) increases the risk of developing psychological issues and social impairments. This hinders the development of their social interaction skills and social-adaptation abilities, leading to a persistent decline in their level of interpersonal security. Therefore, targeted early interventions should be prioritized for early adolescents exhibiting the “low-declining group” interpersonal security development trajectory type, with full attention paid to their social interaction issues. These adolescents should also be provided with greater care, tolerance, support, and understanding through the establishment of a collaborative education mechanism involving families, schools, and society, enabling their sense of interpersonal security to “return” to a normal developmental trajectory.

### 4.2 Influence of interpersonal security development trajectory type on physical activities in school among early adolescents

Multiclass independent variable regression analysis confirmed that the development trajectory of interpersonal security can affect early adolescents’ physical activities in school 1 year later and, the better the type of development trajectory, the more active adolescents’ physical activities in school. This finding deepens research in this field and aligns with some previous perspectives (Engels et al., 2002; Li et al., 2020; Wang et al., 2025). Previous studies have predominantly employed cross-sectional surveys, examining the impact of varying levels of interpersonal security on adolescent behavior at a single point in time, and these studies widely acknowledge that strong interpersonal security reflects secure attachment relationships and being satisfied with relationship needs. Building upon this foundation, individuals can internalize external motivation; strengthen intrinsic motivation; and exhibit positive, active behavioral patterns (Scott et al., 2011; Wang et al., 2025). This study reveals from a dynamic developmental perspective that, during early adolescence, not only does a strong sense of interpersonal security readily foster positive physical activities in school but also that the different developmental trajectory types of interpersonal security exhibit heterogeneity in their influence on physical activities in school. Specifically, compared to adolescents with the “low-declining group” interpersonal security development trajectory type, those in the medium-sustained group exhibited relatively higher levels of physical activities in school. Meanwhile, adolescents in the high-elevated group demonstrated even higher levels of physical activities in school. This aligns with Sullivan’s interpersonal relationship theory, which posits that interpersonal relationships are not static structures but instead a dynamic system. Children and adolescents can develop corresponding social behaviors within these ever-changing and evolving relationships (Sullivan et al., 1953; Sullivan et al., 2017). In other words, the sense of interpersonal security experienced by early adolescents develops dynamically in response to shifts in social status (i.e., acceptance) or emotional connection (i.e., friendship) within interpersonal relationships. The more positive the developmental trajectory, the more likely it is to strengthen self-esteem and self-confidence, fostering proactive engagement in physical activities in school. Conversely, the more negative the developmental trajectory, the greater the likelihood of fostering feelings of inferiority, helplessness, and loneliness, leading individuals to consciously withdraw from group-based, physically and mentally beneficial social activities (e.g., physical exercise).

Social support theory posits that emotional support within the social environment serves as the source for individuals to maintain healthy leisure activities (Hupcey, 2010). Based on this, the present study suggests that fostering a harmonious social interpersonal environment and developing adolescents’ social skills and adaptability during early adolescence can enhance interpersonal security. This helps facilitate either a “stable and positive” or “continuously increasing” development trajectory type of interpersonal security. Consequently, adolescents can maintain positive and pleasant emotions and engage in active physical activities during their school times.

### 4.3 Mediating effect of control beliefs

Multivariate regression analysis showed that, relative to the developmental trajectory type of interpersonal security of the low-decreasing group, the high-elevated group and the medium-sustained group can indirectly promote physical activities in school by enhancing early adolescents’ control beliefs. This finding indicates that, as a form of cognitive control or cognitive response, control beliefs serve as a mediating factor through which developmental trajectories of interpersonal security influence physical activities in school among early adolescents. The theory of control beliefs posits that early adolescents tend to employ locus-of-control strategies (secondary control) more frequently to cope with various stressful events (e.g., peer conflicts and family pressures) (Heckhausen and Schulz, 1995; Spector et al., 2004). Dong and Mao (2018) and Murray (2013) observed that control beliefs serve as a motivating factor for improving adolescents’ physical activity levels, playing a significant mediating role between interpersonal factors and overt behaviors. Unlike previous cross-sectional studies, this longitudinal investigation reveals that dynamically maintaining a good sense of interpersonal security or developing the interpersonal security (specifically, sustaining moderate levels or consistently achieving high levels) helps meet the relational and security needs of early adolescents. Moreover, by enhancing self-confidence and self-esteem, it also fosters control beliefs, energizing adolescents during their school years and promoting physical activity. Broadly, the better the developmental trajectory of interpersonal security, the stronger early adolescents’ control beliefs and, consequently, the more active, proactive, and frequent their physical activities in school.

In conclusion, interpersonal security is a dynamic, subjective experience that can change through interpersonal communication, interaction, and emotional connection. Developing interpersonal security enables adolescents to gain greater emotional support and self-esteem maintenance while stimulating their control beliefs. This enhances self-awareness and improves behavioral states. Conversely, if adolescents perceive threats or experience insecurity in interpersonal interactions, such diminishes their sense of self-mastery and control beliefs, and then they may exhibit negative, apathetic, and withdrawn behavioral tendencies and manifestations during their school years. This aligns with the findings of Richardson and Waite (2002), who concluded that control beliefs are a type of cognitive control of dominant behavior that enable individuals to adjust their behaviors in response to interactions with the external environment. In short, the better the development trajectory type of interpersonal security, the stronger early adolescents’ control beliefs, then the higher their levels of physical activities in school. Therefore, enhancing early adolescents’ social-adaptation skills and fostering interpersonal security may serve as an intervention strategy to cultivate adolescents’ control beliefs and promote their physical activities in school.

### 4.4 Limitations

Some limitations of this study exist and need to be mentioned. First, a three-phase survey spanning an entire year was an insufficient method to clarify the non-linear characteristics of interpersonal security development trajectories. Future studies should employ multiple measurements over several years to accurately trace the dynamic developmental trajectory of interpersonal security. Second, this study focused primarily on adolescents in junior high school, and more research is needed to explore whether the results apply to other samples, such as younger children or undergraduates. Despite these limitations, the present study reinforces previous research by revealing the mediating mechanisms between interpersonal security developmental trajectories and early adolescents’ physical activities in school.

## 5 Conclusion

This study found that early adolescents’ interpersonal security could be divided into three development trajectory types—namely, high-elevated (51.24%), medium-sustained (43.45%), and low-decreasing (5.32%). Relatively, the better an early adolescent’s development trajectory of interpersonal security, the easier it is to enhance their control beliefs and their engagement with physical activities in school. This study also illustrates that educators and parents should pay more attention to the cultivation of early adolescents’ social adaptability and interpersonal communication skills.

## Data Availability

The raw data supporting the conclusions of this article will be made available by the authors, without undue reservation.
